# Does Maintenance of Pulmonary Blood Flow Pulsatility at the Time of the Fontan Operation Improve Hemodynamic Outcome in Functionally Univentricular Hearts?

**DOI:** 10.1007/s00246-021-02599-w

**Published:** 2021-04-19

**Authors:** K. Kalia, P. Walker-Smith, M. V. Ordoñez, F. G. Barlatay, Q. Chen, H. Weaver, M. Caputo, S. Stoica, A. Parry, R. M. R. Tulloh

**Affiliations:** Department of Congenital Heart Disease, University Hospitals Bristol and Weston NHS Foundation Trust, Upper Maudlin Street, Bristol, BS2 8BJ UK

**Keywords:** Fontan, Congenital heart surgery, Pulmonary vascular resistance

## Abstract

It is unclear whether residual anterograde pulmonary blood flow (APBF) at the time of Fontan is beneficial. Pulsatile pulmonary flow may be important in maintaining a compliant and healthy vascular circuit. We, therefore, wished to ascertain whether there was hemodynamic evidence that residual pulsatile flow at time of Fontan promotes clinical benefit. 106 consecutive children with Fontan completion (1999–2018) were included. Pulmonary artery pulsatility index (PI, (systolic pressure–diastolic pressure)/mean pressure)) was calculated from preoperative cardiac catheterization. Spectral analysis charted PI as a continuum against clinical outcome. The population was subsequently divided into three pulsatility subgroups to facilitate further comparison. Median PI prior to Fontan was 0.236 (range 0–1). 39 had APBF, in whom PI was significantly greater (median: 0.364 vs. 0.177, Mann–Whitney *p* < 0.0001). There were four early hospital deaths (3.77%), and PI in these patients ranged from 0.214 to 0.423. There was no correlation between PI and standard cardiac surgical outcomes or systemic oxygen saturation at discharge. Median follow-up time was 4.33 years (range 0.0273–19.6), with no late deaths. Increased pulsatility was associated with higher oxygen saturations in the long term, but there was no difference in reported exercise tolerance (Ross), ventricular function, or atrioventricular valve regurgitation at follow-up. PI in those with Fontan-associated complications or the requiring pulmonary vasodilators aligned with the overall population median. Maintenance of pulmonary flow pulsatility did not alter short-term outcomes or long-term prognosis following Fontan although it tended to increase postoperative oxygen saturations, which may be beneficial in later life.

## Introduction

In the current era, the standard surgical pathway for functional univentricular heart (fUVH) palliation comprises a sequence of three-staged interventions [1] to create a total cavopulmonary connection (TCPC), transforming the prognosis for children with complex fUVH [2]. An incremental improvement in survival has been matched by impressive late functional outcomes, with over 90% of survivors remaining in New York Heart Association (NYHA) classes I–II at follow-up [3–5]. Despite this success, the inherent limitations of this circuit are becoming increasingly pronounced as the population of survivors grows, with substantial disease burden [6, 7] and a decline in freedom from late adverse events [8].

The absence of pulsatile pulmonary perfusion markedly deranges systemic hemodynamics. This paradoxical state of systemic venous hypertension and chronic low cardiac output [9, 10] has pernicious effects on multiple organ systems [11], mediating a progressive, and frequently indolent, circulatory demise—the Fontan ‘paradox’ [10]. There is no universally efficacious treatment for the failing Fontan, aside from targeted surgical or catheter-based interventions for isolated problems. Thus, cardiac transplantation represents the only viable definitive long-term option; this is far from a practical solution with a shortage of donor allografts and the high morbidity and mortality that follows a previous Fontan procedure [12]. As a result, we need to develop novel therapies to effectively manage long-term complications and minimize the inherent inefficiencies of the Fontan circuit.

Total pulmonary vascular resistance (PVR) is a primary determinant of cardiac output after the Fontan procedure [13–15]. As PVR increases, a functional decline in the Fontan circulation is expected [16]. In turn, low PVR is contingent on good pulmonary artery (PA) dimensions [17], a well-developed and compliant pulmonary vascular bed [18], along with other factors such as good systemic ventricular mechanics. There is evidence to suggest that elevated PVR after Fontan may reflect inadequate preparation during earlier staging operations [19].

During the interim bidirectional Glenn (BDG) procedure, a clinical decision is often made whether to exclude or preserve additional sources of pulmonary flow [20]. Currently, conflicting data exist regarding the potential utility or harmfulness of maintaining forward flow through a patent (banded, or natively stenotic) pulmonary outflow tract [21–23]. This anterograde pulmonary blood flow (APBF) may be an important trophic stimulus for the pulmonary arterial tree, with reports of improved PA growth where APBF is preserved [24–27]. However, concerns remain that accessory pulmonary flow may compromise ventricular volume unloading [28], thereby mitigating some of the advantageous changes mediated by the classical BDG [29].

Chronic privation of pulsatile pulmonary flow is detrimental for endothelial function, capillary recruitment, and pulmonary vascular development, all which impact PVR, as seen in both canine [30] and rabbit [31] models. Thus, the argument for preserving pulsatile pulmonary flow is compelling. However, we do not yet know whether this leads to improved hemodynamic outcome in Fontan patients. We have previously shown the utility of maintaining residual APBF following BDG [32], but there is no evidence of studies which correlate this with clinical outcome after the Fontan. We, therefore, hypothesized in the present study that pulsatile perfusion is integral for the maintenance of a compliant and low resistance pulmonary vascular bed.

## Methods

We conducted a single-center retrospective study, involving serial follow-up of each patient who underwent Fontan palliation at University Hospitals Bristol NHS Foundation Trust. Demographic data, clinical data, including primary anatomic diagnoses, cardiac surgical history, inpatient reports, and relevant cardiac investigations, were retrieved from electronic medical records. Formal ethical committee approval was waived given the retrospective anonymized study design, and written informed consent was not required.

### Patient Selection

From January 1999 to December 2018, 183 patients underwent Fontan completion at our institution. Following exclusion of patients with incomplete cardiac catheterization data (*n* = *29*), and those lost during follow-up from other centers (*n* = *48*), 106 patients were included in the analysis.

### Evaluation of Pulmonary Artery Pulsatility

Pulmonary hemodynamics were evaluated using routine preoperative cardiac catheterization data. Transpulmonary gradient (PA mean pressure − left atrial pressure) was recorded and a pulmonary artery pulsatility index (PI) calculated for each patient, as follows:

*PI* = *(systolic pressure − diastolic pressure)/mean pressure*, where all values pertain to PA indices.

The study population was subsequently divided into three, based on pulsatility magnitude. The ‘low PI’ subgroup included patients with PI value < 0.334, ‘medium PI’ between and including 0.334–0.666, and ‘high pulsatility’ > 0.666.

Pulmonary artery size was not routinely measured at our centre during the time period of this retrospective study, and, accordingly, Nakata indices have not been included. Similarly, data concerning the presence or absence of pulmonary collaterals were sparse in available documentation and have consequently not been recorded in this current analysis.

### Clinical Outcome Measures

Standard cardiac surgical outcomes included in-hospital mortality, postoperative ventilation time, duration of chest tube drainage, length of pediatric intensive care unit stay (PICU) and total hospital stay, systemic arterial oxygen saturation (SaO_2_), and medications at discharge.

Longitudinal follow-up included assessment of ventricular function, graded as per echocardiography: 1, severely reduced; 2, moderately reduced; 3, mildly reduced; 4, acceptable or preserved; and 5, normal or excellent. Similarly, atrioventricular valve regurgitation (AVVR) was graded as 1, severe; 2, moderate regurgitation; 3, mild regurgitation; 4, trivial regurgitation; 5, no regurgitation.

Exercise tolerance was graded using the NYHA functional classification, or Ross for children which provides a NYHA-equivalent score [33]. Oxygen saturation by pulse oximetry (at 1-year and last follow-up) and medication support (diuretics and vasodilatory therapy) were also recorded. Intermediate (within one year) and late (after one year) Fontan failure was defined as follows: (i) Fontan takedown or pathway revision, (ii) transplantation, and (iii) in-hospital or Fontan complication-associated mortality, as adapted from The Society of Thoracic Surgeons’ criteria [34, 35].

### Statistical Analysis

Data were analyzed using GraphPad Prism v.8.4.1 software. Two distinct analytical approaches were adopted: (i) Spectral analysis, where PI was charted as a continuum against clinical outcome data, and (ii) subgroup analysis, based on relative pulsatility (low, medium, or high), to identify any non-linear relationships between PI and the defined outcomes measures. Categorical variables are expressed as frequency (%). Continuous data are summarized as either mean ± standard deviation (SD) or median and range values, after testing for normality (Shapiro–Wilk) and equality of variance (*F*-statistic).

For spectral analysis, the Pearson (continuous data) and Spearman (categorical data) rank coefficients (*r*) were calculated to assess correlation between PI and outcome measures. Analysis of variance (ANOVA) (parametric) and Kruskal–Wallis (non-parametric) tests were employed for PI subgroup analysis, as appropriate. All tests were two tailed, and statistical significance accepted for *p*-value < 0.05.

## Results

### Baseline Patient Characteristics

106 subjects were included in our final analysis, of which 66 were male (62.3%). The most common diagnosis was double-inlet left ventricle (18%). Patient demographics and principal diagnoses are summarized in Table 1.

**Table 1:** Patient demographics, palliation history, and baseline assessment

Characteristic	*n* patients (% of subgroup)			*p*-value
	Low PI *n* = *76*	Medium PI *n* = *19*	High PI *n* = *11*	
Gender				0.307
Male	47 (61.8%)	14 (74.7%)	5 (45.5%)	
Female	29 (38.2%)	5 (26.3%)	6 (54.5%)	
Primary diagnosis				
DILV	13 (17.1%)	5 (26.3%)	2 (18.2%)	0.658
HLHS	14 (18.4%)	0 (0.00%)	1 (9.09%)	0.107
TA	10 (13.2%)	2 (10.5%)	3 (27.3%)	0.405
PA IVS	9 (11.8%)	3 (15.8%)	0 (0.00%)	0.410
DORV	6 (7.89%)	2 (10.5%)	2 (18.2%)	0.546
Complex PA	9 (11.80%)	0 (0.00%)	0 (0.00%)	0.146
Complex CCTGA	7 (9.2%)	1 (5.26%)	0 (0.00%)	0.512
Unbalanced AVSD	4 (5.26%)	0 (0.00%)	1 (9.09%)	0.485
MA	1 (1.32%)	1 (5.26%)	2 (18.2%)	0.0224
TGA, SAVV, PS	2 (2.63%)	1 (5.26%)	0 (0.00%)	0.693
Other, complex	2 (2.63%)	3 (15.8%)	0 (0.00%)	0.0407
Age at first palliation	0.0342 (0.00–5.53)	0.0521 (0.00–4.46)	0.466 (0.0137–11.7)	0.0255
Initial palliation type				
Modified BTS	30 (39.5%)	6 (31.6%)	1 (9.09%)	0.137
PAB	15 (19.7%)	9 (47.4%)	3 (27.3%)	0.0479
BTS + PAB	3 (3.95%)	1 (5.26%)	0 (0.00%)	0.760
Norwood	15 (19.7%)	1 (5.26%)	0 (0.00%)	0.0991
BDG	12 (15.8%)	2 (10.5%)	5 (45.5%)	0.0379
Atrial septectomy	1 (1.32%)	0 (0.00%)	2 (18.2%)	0.0052
Age at BDG	0.730 (0.140–11.4)	1.480 (0.397–4.71)	0.874 (0.468–11.7)	0.0077
Fontan assessment				
Pulsatility index	0.167 (0.00–0.333)	0.438 (0.348–0.615)	0.750 (0.667–1.00)	< 0.0001
PASP (mmHg)	12 (6–17)	13 (7.5–21)	16 (11–21.7)	0.0001
PADP (mmHg)	10 (5–16)	8.5 (5–14.5)	7 (5–10.3)	0.0012
LAP (mmHg)	6 (3–13)	6 (4–10)	6 (4–9)	0.263
TPG (mmHg)	3.97 ± 1.93	5.01 ± 2.44	4.73 ± 2.41	0.116
APBF	19 (25%)	11 (57.9%)	9 (81.8%)	0.0002
Pulmonary artery SaO_2_ (%)	75 (61–90)	77.5 (60–93)	79 (66–90)	0.117
Mixed venous SaO_2_ (%)	73.3 (59.3–82.8)	70 (62–81.8)	72.8 (59.8–78.3)	0.782
Systemic SaO_2_ (%)	81 (57–92)	84 (75–89)	79.5 (70–88)	0.211
Age at Fontan	4.61 (1.83–15.3)	5.26 (3.66–17.6)	4.85 (2.56–13.2)	0.101
Fenestration	10 (13.2%)	1 (5.26%)	0 (0.00%)	0.299

*APBF* anterograde pulmonary blood flow, *AVSD* atrioventricular septal defect, *BDG* bidirectional Glenn, *BTS* Blalock–Taussig shunt, *CCTGA* congenitally corrected transposition of the great arteries, *DILV* double-inlet left ventricle, *DORV* double-outlet right ventricle, *HLHS* hypoplastic left heart syndrome, *IVS* intact ventricular septum, *LAP* left atrial pressure, *MA* mitral atresia, *PA* pulmonary atresia, *PAB* pulmonary artery band, *PADP* pulmonary artery diastolic pressure, *PASP* pulmonary artery systolic pressure, *PI* pulsatility index, *PS* pulmonary stenosis, *SaO*_*2*_ oxygen saturation, *SAVV* straddling atrioventricular valve, *TA* tricuspid atresia, *TGA* transposition of the great arteries, *TPG* transpulmonary gradient

The median age at Fontan completion was 4.90 years (range 1.83–17.6 years). Prior procedures included systemic-to-PA shunt (*n* = 37, 34.9%) and pulmonary artery banding (*n* = 27, 25.4%), with four patients (3.77%) undergoing both shunt and banding during initial palliation. 27 patients had a natively stenosed pulmonary outflow tract to protect the pulmonary circuit from volume overload, and bidirectional Glenn (BDG) served as the initial palliation in 15 of these patients. Although we included the hypoplastic left heart syndrome (HLHS) group, it is clear that none of these will have had pulsatile forward flow.

All patients underwent BDG prior to TCPC, at a median age of 0.822 years (range 0.140–11.7 years). Maintenance of anterograde flow was considered on a case-to-case basis. While there was no specific protocol in place, residual forward flow was occluded where there was any indication of volume overloading of the systemic ventricle; this clinical decision required multidisciplinary input and close communication between the medical and surgical teams. At the time of Fontan, 39 patients (36.8%) had residual APBF, but none after the Fontan operation, and a fenestrated Fontan was performed in 11 cases (10.4%). Pre-operative echocardiography showed excellent or preserved ventricular function in 88.5%; however, 54.5% had evidence of at least mild AVVR. Median SaO_2_ at preoperative assessment was 81% (range 57–92%).

### Cardiac Catheterization Data

The median pulsatility index (PI) prior to Fontan completion was 0.236 (range 0–1). Notably, as expected, PI was significantly greater in individuals with persistent APBF versus those without (median PI: 0.364 vs. 0.177), as per Mann–Whitney U test (*U* = 599.5, *p* < 0.0001).

Patients were stratified into three groups according to PI, as follows: low (PI < 0.334, *n* = *76*), medium (0.334 ≤ PI < 0.667, *n* = *19*), and high (PI ≥ 0.667, *n* = *11*) pulsatility. Of the 11 patients who underwent Fontan with fenestration, 10 (90.9%) were in the low PI subgroup.

The mean TPG was 4.23 mmHg (± 2.10), and this somewhat tended to increase with PI. (Pearson *r* = 0.166, *p* = *0.0921* = non-significant). Subgroup analysis did not delineate any significant difference in TPG between the three PI groups (ANOVA, *p* = *0.116*).

### Early Outcomes

There were four early hospital deaths following the Fontan operation, with an early mortality of 3.77%. PI ranged between 0.214 and 0.423 in these subjects. Table 2 outlines the dominant cardiac diagnosis and cause of death.

**Table 2:** Early hospital deaths: key patient characteristics

PI	Age at death	Dominant diagnosis	Cause of death
0.214	6.98 years	TGA, PS and SAVV	Persistent atrial tachyarrhythmias, renal failure and cardiac arrest
0.263	4.54 years	Complex CCTGA	Posterior pericardial effusion leading to cardiogenic shock
0.385	7.36 years	DILV	Severe postoperative bleeding in right chest and cardiac arrest
0.423	14.7 years	Multiple VSDs and RV hypoplasia	Sepsis: cause and source undetermined by cultures

*CCTGA* congenitally corrected transposition of the great arteries, *DILV* double-inlet left ventricle, *PI* pulsatility index, *PS* pulmonary stenosis, *RV* right ventricle, *SAVV* straddling atrioventricular valve, *TGA* transposition of the great arteries, *VSD* ventricular septal defect

**Fig. 1:**
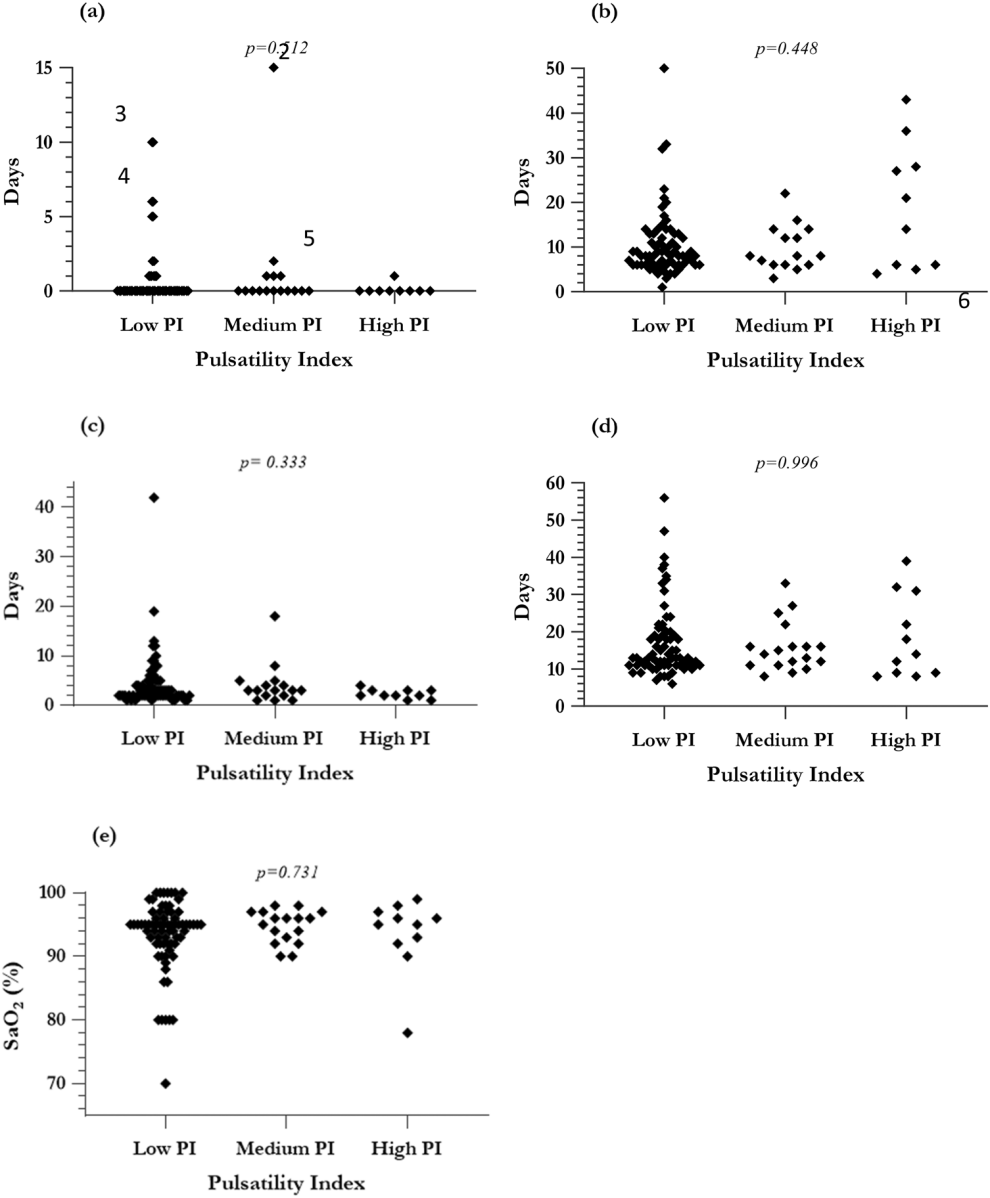
Analysis of standard surgical outcome measures as a function of pulsatility. Results from subgroup analysis are shown, with Kruskal–Wallis *p values*; **a** Ventilation time, **b** Chest drainage duration, **c** PICU stay, **d** Total hospital stay, **e** SaO_2_ at discharge. Abbreviations—*PI* pulsatility index; *PICU* pediatric intensive care unit; *SaO*_*2*_ systemic arterial oxygen saturation

There was no correlation between PI and any of the standard cardiac surgery outcome measures (Fig. 1) including (a) ventilatory time, (b) duration of chest drainage, (c) PICU, and (d) total hospital stay, as per Pearson *r*. Although there were some trends, there was no statistically significant difference for the spectral analysis of ventilatory time *r* = − 0.0948, *p* = 0.351, for duration of chest drainage *r* = 0.123 *p* = 0.229, for PICU stay *r* = − 0.0984 *p* = 0.333, for total hospital stay *r* = − 0.0386 *p* = 0.697, for oxygenation at discharge *r* = 0.0794 *p* = 0.430. This remained consistent on subgroup analysis, as described below.

The median SaO_2_ at discharge was 95% (range: 70–100%). As expected, this was significantly higher than at preoperative assessment (Mann–Whitney U = 499, *p* < *0.0001*). There was no correlation between PI and SaO_2_ at discharge (Pearson *r* = 0.0794, *p* = *0.4301*), and no notable inter-group differences either (Kruskal–Wallis, *p* = *0.1159*) (Fig. 1e). Two patients with fenestrated Fontan had complicated postoperative courses, one due to acute ischaemic stroke and one due to prolonged bilateral pleural effusions. However, upon comparing median values, outcomes of those with fenestration were congruent with the remainder of the low PI subgroup.

### Long-Term Outcomes

The median follow-up time was 4.33 years (range 0.0273–19.6 years). No further deaths were reported, with an overall survival of 93.6% in this cohort.

Five patients (4.72%) developed complications within the first year, three of whom required surgical or catheter-based intervention; one developed severe congestive heart failure at seven months, necessitating cardiac transplantation. The two others developed Fontan failure at two months postoperatively, due to (i) complete occlusion of the left pulmonary artery, managed by balloon dilatation with stenting, and (ii) right ventricular dysfunction secondary to arrhythmia, requiring fenestration. Table 3 summarizes the pertinent clinical information of these cases.

**Table 3:** Intermediate-term Fontan failure, key clinical information of affected patients

PI	Time after Fontan	Dominant diagnosis	Complication
0.00	3 months	Unbalanced AVSD	Pleural effusion
0.0909	6 months	Unbalanced AVSD	Plastic bronchitis
0.125	2 months	Complex CCTGA	Complete occlusion of LPA
0.286	2 months	HLHS	RV dysfunction, secondary to frequent VEBs
0.692	7 months	Unbalanced AVSD	Severe congestive heart failure

*AVSD* atrioventricular septal defect, *CCTGA* congenitally corrected transposition of the great arteries, *HLHS* hypoplastic left heart syndrome, *LPA* left pulmonary artery, *PI* pulsatility index, *RV* right ventricle, *VEB* ventricular ectopic beat

At last follow-up, echocardiographic assessment revealed excellent or preserved ventricular function in 92.7% and at least mild AVVR in 55.5%. Changes in ventricular function and AVVR were calculated as the net difference between preoperative and most recent echocardiograph gradings. There was no linear correlation relationship between PI and changes in ventricular function or AVVR. The median change in ventricular function and AVVR in all three PI subgroups was 0; however, those with medium PI tended towards improved ventricular function (Kruskal–Wallis, *p* = 0.0723), but this trend was not replicated with AVVR. (Table 4). MRI data were available for 55 patients (51.9%) and were reported as either left ventricular indices (*n* = *24*), right ventricular indices (*n* = *12*) or combined left and right ventricular indices (*n* = *19*). Given this incomplete and heterogeneous dataset, formal analysis was not completed.

**Table 4:** Spectral and subgroup analysis of categorically graded late outcome data

Outcome measure	Spearman *r*	*p*-value	Kruskal–Wallis *p*-value
Change in ventricular function	0.0379	0.714	0.0723
Change in AVVR	− 0.126	0.211	0.399
Exercise tolerance	− 0.124	0.227	0.459

*AVVR* atrioventricular valve regurgitation

**Fig. 2:**
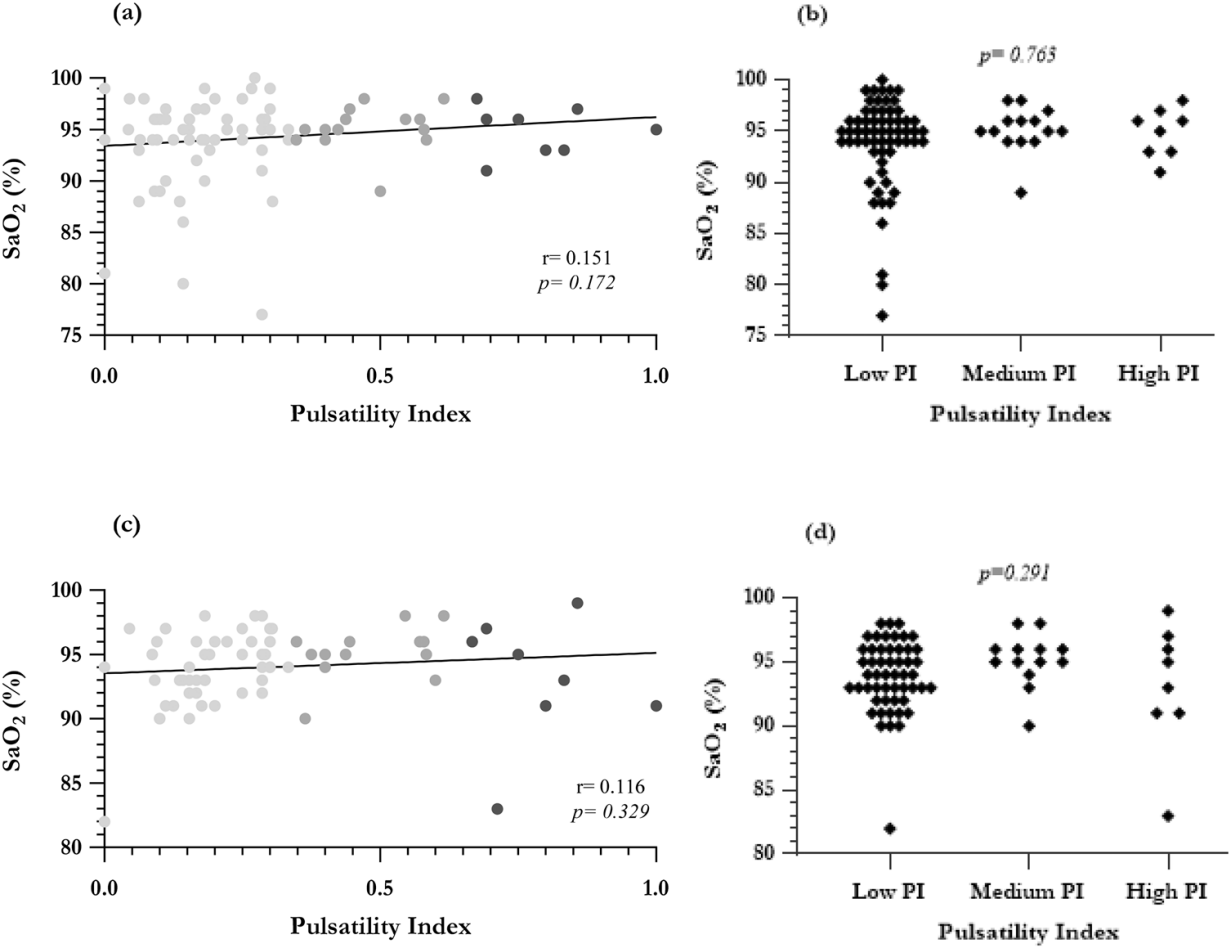
Long-term oxygenation status at 1-year and last follow-up. Spectral analysis is presented on the left-hand side, at **a** 1-year and **c** last follow-up, with calculation of Pearson *r* and associated *p* value. Light gray represents data points from the ‘low PI,’ medium gray from the ‘medium PI,’ and dark gray from the ‘high PI’ group. On the right, results from subgroup analysis are shown, at **b** 1-year and **d** last follow-up, with Kruskal–Wallis *p values*

The cohort demonstrated good exercise tolerance during follow-up, with 96.9% falling into class I–II of the NYHA or Ross classification. Formal analysis of the CPET data was not performed as the data were limited (*n* = *19*, 17.9% of cohort). At 1-year follow-up, the median systemic arterial oxygenation was 95% (range 77–100%). In patients who had completed more than one postoperative year, the last recorded SaO_2_ was 95% (range 82–100%) at a median of 6.3 years follow-up (range 1.19–17.77 years). While there was a tendency toward improved oxygenation with increased pulsatility, this was not statistically significant on spectral or subgroup analysis (Fig. 2). Clinical outcomes of those with fenestrated Fontan aligned with the remainder of the low PI subgroup and with the overall population median.

During the follow-up period, a further 11 patients (13.2%) developed Fontan-associated complications (Table 5). These included intractable arrhythmias (atrial, *n* = 3; ventricular, *n* = 1), Fontan-associated liver disease (*n* = 4), protein-losing enteropathy (*n* = 2), and arterial collaterals (*n* = *1*). Of note, two patients who developed atrial arrhythmias had underwent fenestrated Fontan. The median time at onset of late complications was 6.06 years; however, four patients out of 11 (36.4%) developed complications at or after 10 years.

**Table 5:** Intermediate and late complications following the Fontan operation, key clinical information

Complication	Pulsatility index	Time after Fontan (years)	Primary cardiac diagnosis
Atrial arrhythmia	0.2	6.06	DORV
Atrial arrhythmia	0.2	14.8	DILV
Atrial arrhythmia	0.3	2.19	Unbalanced AVSD
Ventricular tachycardia	0.154	1.72	PA IVS
FALD-FNH	0.25	10.7	TA
FALD-splenomegaly	0.304	9.64	DILV
FALD-cirrhosis	0.4	16.1	DILV
FALD-fibrosis	0.579	13.8	TA
PLE	0.176	1.45	HLHS
PLE	1	2.44	DILV
Arterial collaterals	0.0645	2.37	DORV

*AVSD* atrioventricular septal defect, *DILV* double-inlet left ventricle, *DORV* double-outlet right ventricle, *FALD* Fontan-associated liver disease, *FNH* focal nodular hyperplasia, *HLHS* hypoplastic left ventricle, *PA IVS* pulmonary atresia with intact ventricular septum, *PLE* protein-losing enteropathy

**Fig. 3:**
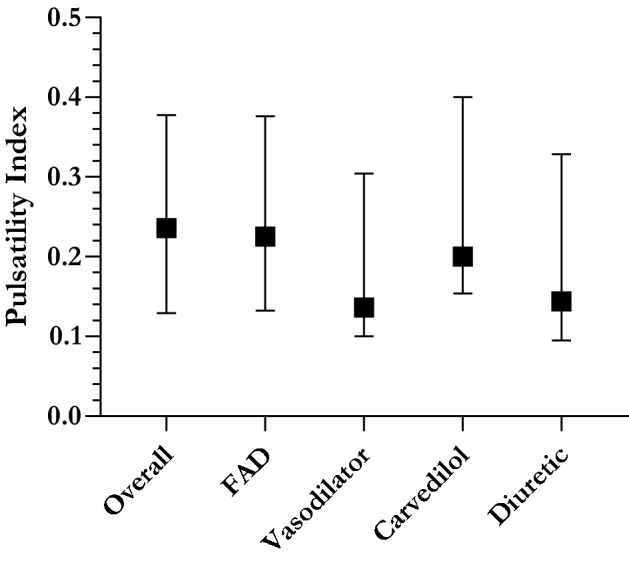
PI and adverse long-term outcomes. Distribution of Pulsatility Index (PI) in Patients with Fontan-associated complications, and those requiring pulmonary vasodilator or diuretic therapy at last follow-up. Median and interquartile range (IQR) are plotted. Abbreviations—*FAD* Fontan-associated disease or complications; *PI* pulsatility index

The median PI in those with intermediate- or late-Fontan-associated complications (*n* = *16*, PI = 0.225) and those receiving (i) pulmonary vasodilator therapy (*n* = *7*, PI = 0.1364), (ii) carvedilol (*n* = *7*, PI = 0.20), or (iii) diuretics (*n* = *10*, PI = 0.144) largely aligned with the overall population median (0.236), and included individuals from all three (low, medium, and high) PI subgroups (Fig. 3). While the absolute median PI values were lower in these patient subsets, the event rate was too small for statistical significance.

Of those with fenestrated Fontan, three patients (27.2%) were prescribed carvedilol in the long term, and one prescribed both carvedilol and diuretics (9.09%), indicating a significant associated morbidity.

## Discussion

Despite its unprecedented success, the inherent haemodynamic aberrancies of the Fontan circulation align with a time-related decrement in functional status [36], an ongoing risk of severe complications, and a life expectancy that remains significantly lower than the biventricular population [37]. Crucially, impaired pulmonary endothelial function, due to loss of pulsatile perfusion, may play a dominant pathophysiologic role in the development of elevated PVR in Fontan patients. We have, however, shown that maintenance of pulmonary artery pulsatility with some form of forward flow at the time of the Fontan does not alter the short-term outcomes, or long-term prognosis but is associated with increased postoperative oxygen saturations.

The benefits of pulsatile blood flow are prominent in the literature. In lungs isolated from neonatal rabbits, increased pulsatility levels mediated a reduction in vascular resistance [38]. This finding has been corroborated in a chronic porcine model of BDG [39], where partial ligation of the PA (micropulsatile group with APBF) was associated with a significantly lower PVR, compared to animals who underwent complete PA ligation. Notably, flow pulsatility positively correlated with maximal vasodilatory response to acetylcholine, and attenuated the development of pulmonary hypertension; this implies a direct role of pulsatility in preserving endothelial function and demonstrates the underlying functional sensitivity of the vascular endothelium to shear stress magnitude [40].

This prompts several considerations for our study: first, the need to determine the optimal level of auxiliary APBF to both minimize risk of ventricular volume overload and, concurrently, retain sufficient pulsatility to preserve pulmonary endothelial function. To complicate matters further still, this figure is unlikely to remain static, given the continued somatic growth of the child during the palliation process. Next, where pulmonary artery banding restricts anterograde outflow to subcritical levels, blood flow becomes increasingly laminar in character. Similarly, hemodynamics resulting from a systemic-to-PA shunt, or where significant aortopulmonary collaterals have developed [41], may more closely mirror the arterial, rather than venous, waveform and possess a pulsatile character. On this premise, our study design refrained from arbitrarily classifying subjects as ‘pulsatile’ and ‘nonpulsatile’ based on preconceived notions regarding APBF. Nevertheless, analysis of our cohort found that pulsatility index was significantly greater in those with residual anterograde flow (*p* < 0.0001).

This present study did not elicit any significant differences in early or late clinical outcomes based upon PA pulsatility at the time of Fontan. Although the median PI in those requiring long-term vasodilator or diuretic therapy was notably lower than the cohort average (0.136, 0.144, and 0.236, respectively), the event rate was too low for robust statistical analysis. One of the main practical challenges of our study design was the retrospective assessment of pulsatility. We elected to use the equation: Pulsatility Index (PI) = (PA systolic pressure–PA diastolic pressure)/PA mean pressure, as adapted from the doppler-derived blood velocity formula which is routinely employed in other clinical fields, including obstetrics [42] and stroke medicine [43]. A literature search identified one previous record of this equation in assessing pulmonary blood flow pulsatility, in a study attempting to differentiate between pulmonary arterial and chronic pulmonary thromboembolic hypertension using PI [44]. We viewed this to be more appropriate and physiologically sound than other indices; in particular, the novel pulmonary artery pressure index (PAPi) = (PA systolic pressure/PA diastolic pressure)/right atrial pressure [45], which holds prognostic relevance in right heart failure, would likely be an unreliable measure in the setting of AVVR, as commonly observed in our patients.

The lack of (i) unanimous criteria to quantify and (ii) a robust method to assess pulsatility is problematic. Other centers have adopted echocardiographic [46] or CMR-based [47] assessments of PA pulsatility index, using the doppler-derived formula alluded to above, where PI = (peak systolic velocity – end diastolic velocity)/time averaged velocity. Echocardiography would likely be technically challenging, due to the acoustic interference from lung tissue, and may be limited by poor reproducibility [48]. However, CMR is a particularly appealing tool with the potential to not only analyze the complex flow dynamics but also provide detailed anatomic and functional information about the single ventricle. Indeed, it has previously been demonstrated that pulsatile flow is dependent on energy, rather than pressure, gradient [49], with pulsatile flow transmitting 2.4 times the amount of energy as nonpulsatile flow at the same mean pressure [50]. As CMR can assess flow velocities, in addition to pressure indices, a prospective study employing MRI-derived PI would likely enable superior quantification of pulsatility: an interesting arena for future-related work.

This study was limited by its retrospective nature. Likewise, the functionally univentricular heart represents a highly heterogeneous group of complex defects, and therefore, the effects of pulsatile pulmonary flow on one specific anatomic substrate might not be generalizable to a morphologically different single ventricle. Inclusion of those patients with HLHS might also introduce bias since none of these would have forward flow at any point. They were retained in order to provide larger groups for comparison, and with larger numbers of patients in each group, they might be omitted in a future study. Analysis of larger subgroups of UVH patients, over a longer timeframe, is necessary to elucidate specific defects which may respond more favorably to pulsatile blood flow; this would ultimately enable evidence-based risk stratification and management.

## Data Availability

This manuscript reports original results. Individual participant data will not be made available at a later date.
